# Immunometabolism and the Kinome Peptide Array: A New Perspective and Tool for the Study of Gut Health

**DOI:** 10.3389/fvets.2015.00044

**Published:** 2015-10-13

**Authors:** Ryan J. Arsenault, Michael H. Kogut

**Affiliations:** ^1^Department of Animal and Food Sciences, University of Delaware, Newark, DE, USA; ^2^United States Department of Agriculture, Agricultural Research Service, Southern Plains Agricultural Research Center, College Station, TX, USA

**Keywords:** immunometabolism, gut health, kinome, immunity, metabolism, animal agriculture

## Abstract

Immunometabolism is a relatively new research perspective, focusing on both metabolism and immunology and the cross-talk between these biological processes. Immunometabolism can be considered from two perspectives; 1) the role that immune cells play in organ metabolism and metabolic disease, and 2) the metabolic processes that occur within immune cells and how they affect overall immunity. The gut may be the prototypical organ of immunometabolism. The gut is the site of nutrient absorption and is a major, if not the major, immune organ. We also describe the integration of kinomics and the species-specific peptide array to the study of the gut. This unique immunometabolic tool combined with the unique immunometabolic nature of the gut provides significant research potential to many animal health applications.

## Immunometabolism

The interface of the immune system and metabolism is an emerging field of study. Relatively recently, immunity and metabolism were treated as distinct processes carried out by an organism. Immunity was focused on the recognition and resistance to a pathogen and involved its own set of cells and tissue activities. Metabolism was solely the chemical processes that provided the energy to carry out the various functions of the organism; this included immune functions, but metabolism was simply the source of energy for the immune system.

The perspective linking immunity and metabolism is styled as immunometabolism. Immunometabolism can be considered from two sides: 1) the role of immune cells in metabolism in organs and the effects on whole organism metabolism or 2) the role of metabolic pathways in immune cells and the effect on broader immunity (1). Early studies in immunometabolism from the first perspective involved human health concerns related to obesity, diabetes, and metabolic disorder (2). Excessive fat deposition can lead to an innate immune inflammatory response. Chronic low-grade inflammation has been linked to metabolic diseases, such as type 2 diabetes, fatty liver disease, and atherosclerosis. Studies typifying the second perspective involved the role of some of the classic metabolic energy sensors and energy switches, such as the signaling proteins AKT1–3, AMPK, mTOR, and LKB1; these were shown to be linked to CD8+ T cell (3) and other immune cell functions. From there, links between metabolism, immunity, and host response to infectious disease grew.

Within animal agriculture, a consideration of immunometabolism in animal production has been ongoing, though not coined as such. It has been clear to poultry producers that a focus solely on maximizing animal growth can be detrimental to immune potential, while an innate immune response has negative consequences on growth (4). Integrating metabolism and immunity provides a research avenue for the ultimate goal of maximizing growth and animal production without having a negative impact on animal health and immunity. Our own research has shown the nearly innumerable links between cellular signaling proteins classically characterized as members of either the immune or metabolic functional groups (5). Due to these links, we feel that an integrated immunometabolic approach is worth considering for anyone researching animal production from either a nutrition/metabolism or immunity/disease perspective. Below, we describe some research categories that fall under the immunometabolism umbrella and their relevance to animal agriculture.

### Growth/Immunity Balance

A significant avenue of research combining immunity and metabolism in animal production was how mounting an immune response affected energy levels and the transfer of energy from growth to immunity (4). Research into the energy consequences of immunity is relatively advanced in animal science. It has been well understood for many decades that an animal that initiates an innate immune/inflammatory response will likely grow slower and have worse feed conversion (6, 7). It is thought that one mode of action of growth-promoting antibiotics given to food animals is a general reduction in inflammation. Indeed, it has been argued that the anti-inflammatory effects of growth-promoting antibiotics are even more important than their reduction/elimination of disease-causing pathogens (8). In human medicine, disease early in life has been linked to ultimate growth potential. Less incidence of disease as infants results in greater growth and ultimate height in adulthood (9).

### Obesity, Inflammation, Immunity, and Metabolism

With growing research into obesity and associated ailments, including metabolic syndrome, diabetes, and heart disease, a new perspective on the interaction of immunity and metabolism emerged (10). It was found that chronic low-level inflammation was a symptom of obesity (11); this inflammation could lead to diseases like diabetes, among other ailments.

In the animal science field, feed-induced inflammation has been a concern. Certain feed ingredients can lead to an inflammatory gut response; examples include non-digestible components of wheat and rye in chickens (12) and soybean meal in fish (13, 14). Even an excess of feed can lead to changes in immune response (15). One current animal feed strategy involves adding enzymes to break down certain indigestible and/or inflammatory feed components in the gut, with the aim to reduce immune response and redirect this energy to growth (16). A current feed trend involves trying to find natural additives that enhance the animal’s resistance to disease, either by influencing the host immune response or the gut microbiota. Caution must be exercised when evaluating these feed additives; robust scientific methodology must be used to determine efficacy and understand the mechanism of action.

In dairy cattle, the transition period immediately before and after calving is an important immunometabolic period. During this time, a dairy cow’s immune functions are impaired, as the mobilization of lipids causes susceptibility to both metabolic and infectious disease (17). This increase in fatty acids (FAs) in the blood can lead to uncontrolled inflammation and oxidative stress. The dysfunction in the inflammatory response, due to the free FAs increase, is the link between metabolism and immunity during the calving period.

In poultry, there has been a significant amount of research into nutrition’s effects on immunity (18) and the use of pre- and probiotic feed ingredients to improve growth and disease resistance (19–21). However, the literature is limited on the immunometabolism link between stress or disease and production issues. The links between disease and production issues are certainly there, and poultry production problems ranging from lameness (22) to muscle fat deposition (23) have been explored.

As discussed in the following section, the study of immunometabolism now incorporates the metabolic pathway changes in immune effector cells, such as macrophages and T-cells that lead to changes in their activation or immune activity. A promising new avenue of research is the targeting of metabolic machinery and metabolic pathways of immune cells as an alternative means of modulating the immune response.

### Intracellular Immunometabolism Interactions

The recent expansion of the immunometabolism field involves characterizing the direct intracellular pathway links between metabolism and immunity (1, 24). Research is focusing on signaling molecules that integrate both metabolic energy sensing and immune response signals; some examples include mTOR, AMPK, and sirtuins. The protein synthesis pathway is regulated by mTOR and is also involved in T-cell fate (25), determining whether the cell becomes an effector T-cell or a regulatory T-cell (3). AMPK is an energy sensor that monitors the ratio of AMP:ATP, altering anabolic and catabolic processes; it is also involved in innate immune response and has a direct link to mTOR (26). Evidence also points to metabolic-induced epigenetic reprograming of immune pathways via the sirtuins (27). The past perspective of separating immunity and metabolism meant a focus on targeting immune pathways in infectious disease and metabolism pathways for growth/metabolic disorders. With an integrated approach, we can broaden our potential targets for disease intervention and our understanding of how metabolic processes can influence health.

### Oxidative Phosphorylation, Glycolysis, Warburg Effect, and Immune Response

Studies of the metabolism of immune cells have shown that metabolic processes determine immune function. In dendritic cells and macrophages, the switch from oxidative phosphorylation (OXPHOS) to aerobic glycolysis, which can be triggered by immune ligands like LPS, leads to profound immune activity changes (24). These changes include a release of proinflammatory cytokines, an increase in cell migration, and the utilization of OXPHOS machinery (28, 29) for the production of immune effectors such as nitric oxide (NO) and reactive oxygen species (ROS) (30, 31). In fact, without the change to aerobic glycolysis initiated by immune ligands, the electron transport chain proteins in the mitochondria would not be available to generate these potent antibacterial molecules. The change in metabolism when immune cells are activated has been described as akin to the Warburg effect in cancerous cells, leading to aerobic glycolysis-fueled proliferation and activity (32).

### T-Cells and Metabolism

In T-cells, the metabolic processes that are activated can help determine the ultimate function of the cell. Effector T-cells undergo active glycolysis, utilizing this energy to carry out immune activities and proliferate. Quiescent T-cells rely on glycolysis, the citric acid cycle, and OXPHOS (1). Memory T cell metabolism is biased toward free FA metabolism, and the proliferative machinery is turned off. This gives memory T-cells a lower metabolic rate and a longer life span, allowing them to survive and circulate longer than effector T-cells (33).

## Immunometabolism and the Gut

The gut is the prototypical organ for considering immunity and metabolism as an integrated whole. The gastrointestinal system is the site of significant food breakdown and nutrient absorption, and it is the largest lymphoid organ in the body secreting the most antibodies in humans (34) and animals (35).

In the production of food animals, the gut is a major focus. Effective and efficient nutrient absorption is the first and major step in cost-effective animal production. With the gut as the site of nutrient absorption, any defect in the ability to extract nutrients from feed can have a profound impact on growth and disease susceptibility. In addition, feed efficiency is of critical importance to animal producers, as higher feed efficiency increases the amount of commodity produced and reduces costs. Any increases in feed efficiency from an animal perspective must take place in the gut.

The gut is a major, if not the major, immune organ. It is the main mucosal immune site, and a majority of a body’s immune tissue and immunoglobulin producing cells are found in the gut. Infections of the gastrointestinal tract can have huge implications on animal health, gut function, meat contamination, and the spread of disease. A large proportion of disease-causing microbes in food animals enter the host via the gut (36). Manipulation of the gut is a multi-billion dollar target for animal industry products, including prebiotics, probiotics, antibiotics, anti-parasitics, feed enzymes, and feed additives, among others. Many of the pathogens that are considered a food safety concern originate or reside in the animal gut. A proper understanding of the gut can lead to more efficient animal production, less disease, and safer food.

The microbiome, the central component of gut physiology, should be considered in any discussion of the gut and immunometabolism. The microbiome is a key nutritional/metabolic component of the gut, as gut microbes break down otherwise indigestible components of food, providing absorbable and further digestible metabolites (37). The microbiome is an immune component of the gut, as the resident microbes are competitors for pathogens that enter the gut (37, 38). Adding microbes or altering the ratio of microbe species in the animal gut to competitively exclude pathogens is undergoing a significant amount of research and development. The commensal microbes are also critical to proper stimulation and development of the neonatal immune system and help the gut immune system to maintain a balance between tolerance and active immune response (38, 39).

## Immunometabolism and the Kinome Peptide Array

### Species-Specific Peptide Arrays for Kinome Analysis

Peptide arrays have become a productive, high-throughput method of studying the active kinome, the kinase complement of a cell or tissue (40). The principle involves immobilized kinase-target peptide sequences printed on a glass array. Exposing the array to lysate containing active kinases, from gut tissue, for example, results in peptide phosphorylation and can generate a visual signal of substrate–enzyme phosphorylation. By comparing the relative signal of experimental cell or tissue samples, one can identify changes in signal transduction pathways and phosphorylation-regulated events.

It is often the case that new, high-throughput methodologies are designed for the standard laboratory species, mice, and rats, or for work with human samples. This was also the case with the kinome peptide arrays. Through extensive research and development, a methodology for designing and using species-specific kinome arrays was developed (41–43). The use of this technology in agricultural species has been reviewed extensively elsewhere (44) and has been used to design peptide arrays to study important biological questions in a number of agriculturally important species, including bovine (45) and poultry (46).

### Immunometabolism Array

Not only are the peptide arrays designed to be species-specific but also they can be process-specific. The initial species-specific arrays were designed to study the innate immune response and contained numerous signaling pathways intermediates involved in this response, such as toll-like receptor (TLR) signaling members, inflammatory intermediates, and others (45). Subsequently, in order to study the metabolic consequences of stress responses, a metabolic peptide array was designed. This array incorporated protein, carbohydrate, and FA metabolic signaling intermediates as well as key energy regulating proteins (23, 47). We have conducted numerous studies on the physiology and host–pathogen interactions in agricultural species using the species-specific peptide arrays. Our analysis of these data showed that the metabolism and immune processes may be distinctions without differences, in that they are two integrated parts of a single cellular process (5). It became clear how much protein–protein interaction there was in the pathways represented on each array. This level of interaction was the impetus for our design of an immunometabolic, species-specific peptide array for both poultry (chicken and turkey) and cattle. This latest generation of species-specific peptide arrays provides an integrated immunometabolic approach to studying kinome response (5). These large arrays, representing approximately 1,000 individual peptides, have been designed to cover the entire network of immunometabolism, including innate and adaptive immunity, protein, carbohydrate, and FA metabolism, as well as hormone and stress response. This integrated peptide array will allow for the study of both the immune and metabolic consequences of an environmental condition, disease, treatment, additive or intervention, and the interactions between them.

In the realm of animal agriculture, nutrition/metabolism and immune performance have been two fields that have been converging for many years. Producers, veterinarians, and animal scientists have come to understand that a sole focus on growth can often come at the expense of health and disease susceptibility, and a strong response to disease can have significant effects on growth. With a tool that can study both metabolism and immunity simultaneously, these two areas of animal science no longer have to be at odds. We can study nutrition and observe effects on immune responses or, conversely, study disease and see how this may effect growth. Our group has already published data that show that *Salmonella* infection of chicken can have effects on the fat deposition and carbohydrate metabolism in peripheral muscle (23). The results indicated that the dysbiosis caused by the *Salmonella* in the gut effected metabolic processes in the skeletal muscle. We are currently working on projects to in which we hope to show an immunometabolic response to infectious diseases and feed-induced inflammation of the gut. This represents only a small fraction of the research potential of this approach.

## Conclusion

The search for antibiotic alternatives in animal production has renewed the research focus on gut health. It seems likely that any effective alternative will center in the gut. Immunometabolism has expanded from the study of chronic, low-level inflammation, and obesity to a full research perspective, encompassing a variety of fields. The adaption of kinomics to animal agriculture is a relatively recent development and has provided valuable insight into animal biology. Integrating gut health, immunometabolism, and kinomics have significant potential in animal production/health, feed additive development, drug discovery, reproduction, and disease research. Here, we have described this new perspective in gut health and animal production research and a useful tool to carry it out.

## Conflict of Interest Statement

The authors declare that the research was conducted in the absence of any commercial or financial relationships that could be construed as a potential conflict of interest.
